# Application of concrete surfaces as novel substrate for immobilization of TiO_2_ nano powder in photocatalytic treatment of phenolic water

**DOI:** 10.1186/s40201-015-0214-y

**Published:** 2015-08-04

**Authors:** Mohammad Delnavaz, Bita Ayati, Hossein Ganjidoust, Sohrab Sanjabi

**Affiliations:** Civil and Environmental Engineering Faculty, Tarbiat Modares University, Tehran, Iran; Civil Engineering Department, Faculty of Engineering, Kharazmi University, Tehran, Iran; Material Engineering Department, Nano Materials Division, Tarbiat Modares University, Tehran, Iran

**Keywords:** Concrete, Immobilization, Intermediates, Water cleaning technology

## Abstract

**Background:**

In this study, concrete application as a substrate for TiO_2_ nano powder immobilization in heterogeneous photocatalytic process was evaluated. TiO_2_ immobilization on the pervious concrete surface was done by different procedures containing slurry method (SM), cement mixed method (CMM) and different concrete sealer formulations. Irradiation of TiO_2_ was prepared by UV-A and UV-C lamps. Phenolic wastewater was selected as a pollutant and efficiency of the process was determined in various operation conditions including influent phenol concentration, pH, TiO_2_ concentration, immobilization method and UV lamp intensity.

**Findings:**

The removal efficiency of photocatalytic process in 4 h irradiation time and phenol concentration ranges of 25–500 mg/L was more than 80 %. Intermediates were identified by GC/Mass and spectrophotometric analysis.

**Conclusions:**

According to the results, photocatalytic reactions followed the pseudo-first-order kinetics and can effectively treate phenol under optimal conditions.

## Background

The ability of Advanced Oxidation Processes (AOP_s_) in treating a wide range of hazardous wastes has brought this technology to the forefront of research over the last decade. Among AOP_s_, application of heterogeneous photo-catalysis by using semiconductors has been proved to be real interest as an efficient tool for degrading both aquatic and atmospheric organic contaminants [1]. Semiconductors are photo-reactive metal oxides for contaminants eradication that refer to photo-catalysts [2]. Titanium dioxide (TiO_2_) is an established photocatalyst utilized in the photo-oxidation process. When TiO_2_ is exposed to the appropriate wavelength of ultra-violet light (UV-A), electrons in the low-energy valence band will absorb the photon’s energy and move into the high-energy conduction band. The result of this electron excitation is a hole, or positive charge, in the valence band (h^+^) and an electron in the conduction band (e^−^) [3]. Reaction yield and photocatalytic activity will be increased when the diameter of TiO_2_ particles becomes smaller especially below 100A° [1].

Photocatalytic reactors for water and wastewater treatment can be classified to slurry and photocatalytic ones. In the slurry reactors, the catalyst particles are freely dispersed in the fluid phase (water) and consequently the photo-catalyst is fully integrated in the liquid mobile phase [4]. Whereas the immobilized catalyst reactor design features a catalyst anchored to a fixed support and dispersed on the stationary phase. Slurry systems require the separation of the fine sub micron particles TiO_2_ from the treated milk-like water suspension. Separation steps cause to complicate the treatment process and decrease the economical viability of the slurry reactor approach [5]. Therefore, these difficulties have led many researchers to study reactors with thin immobilized films of catalyst bonded to a solid substrate such as activated carbon [6], fiber optic cables [7] fiberglass [8], glass beads [9], quartz sand [10], silica gel [11] and stainless steel [12]. Dip coating from suspension, spray coating, sputtering, sol–gel related methods, and electrophoretic deposition [4, 13] are techniques developed for immobilizing TiO_2_ catalysts on these substrates. Although these techniques and substrates had a suitable performance for treating different types of wastewater in laboratory scale experiments, but their application in wastewater treatment plants and pilot scale studies are questionable.

Most applications of concrete modified by TiO_2_ were done for air pollutant removal and self-cleaning surfaces [14]. Although many studies in the field of air pollution have been done by concrete-TiO_2_ photocatalyst process [15, 16], but the number of wastewater treatment researches is very low. Application of concrete as TiO_2_ substrate has unique characteristics such as porosity, natural abundance, absence of toxicity, and low price. This construction material is used in all of the water and wastewater treatment plants (WWTP) all over the world. Therefore, the photocatalytic process by TiO_2_ photocatalyst that immobilized on concrete surfaces can be used in large scale WWTP.

Phenols and its compounds are widely found in paint, leather and textile, disinfectants, medicine, oil refinery and lubricant production wastewater industries in Iran [17–19]. Phenol is rapidly absorbed through the skin and can cause skin and eye burns upon contact. Comas, convulsions, cyanosis and death can be resulted from its overexposure [20, 21]. Therefore Phenol-containing wastewater may not be conducted in open water without treatment because of its toxicity.

Several investigations using different physical, chemical and biological systems and their combination for phenol elimination from wastewater have been reported [22–27]. Biological processes are preferred to other conventional methods due to their ability to effectively destroy the pollutants in an environmentally benign and cost effective way [19]. Sensitive of process to organic shocking load and need to high control for microbial acclimation for hard degradable compound such as phenol are limiting factor in biological treatment. Photocatalytic process didn’t have these limitations and can be used separately or in joint with biological process. Different researches were done related study to photocatytic process in wastewater treatment. Chiou et al. (2008) have studied degradation of phenol and m-nitrophenol using a photocatalytic process in aqueous solution by commercial TiO_2_ powders (Degussa P-25) under UV irradiation [28]. The optimal solution pH of single phenol and m-NP was at around 7.4 and 8.9, respectively. Immobilization of TiO_2_ on perlit granules for photocatalytic degradation of phenol was done by Hosseini et al. (2009) [13]. The Results showed uniform coating on perlit and good photocatalytic activity for the catalysts. Pumic stone was applied as TiO_2_ substrate for degradation of dyes and dye industry pollutants [29]. Real wastewaters collected before biological treatment treated in photocatalytic reactor and color disappeared after 4-h.

The main objective of this research was to study the feasibility of using concrete as substrate for TiO_2_ nano powder immobilization to treat phenolic wastewater. For this reason, four methods were applied for immobilization of nano TiO_2_ on concrete. Kinetic of the reactions, long term use of the process and intermediates formed during the photo degradation were other objectives of this research.

## Material and methods

### Chemicals

Cement in the concrete was an ordinary Portland type V that is usually applied in WWTP construction in Iran. Selection of concrete mix proportion was done according to standard practice for selecting proportions of normal concrete (ACI 211.1 :1996) [30]. Light expanded clay aggregate (LECA) was used as light coarse aggregate to lead specific gravity of concrete to 1200 kg/m^3^. LECA application increased surface porosity of concrete and promoted the specific surface area as an important immobilization parameter. Concrete surface was fabricated in wooden moulds with an internal dimension of 500 × 250 × 50 mm. TiO_2_ Degussa P25, anatase/rutile ca. 70/30, with an average particle size of 20 nm and a BET surface area 55 ± 15 m^2^.g^−1^ was used as photocatalyst. UV radiation was provided by different powers of UV-A Philips medium pressure lamps. The spectral irradiance of UV-A lamp ranges from 300 to 400 nm. The primary wavelength distribution of the UV-A lamp was 365 nm and the light intensity was among 4.42-8.9 mW.cm^−2^. The incident UV-A light intensity was measured by a UV power meter (UVA-365- Lutron Taiwan). Also UV illumination was provided by UV-C lamps (20 Watts, maximal light intensity at 256 nm).

Phenol (purity above 99 %) as a contaminant, NaOH, HCl for pH adjustment, NH_4_OH, K_2_HPO_4_, KH_2_PO_4_, K_3_Fe(CN)_6_ and 4-aminoantiprine as phenol concentration reagents and other chemicals all offered by Merck Co., as an analytical reagent grade. Wastewater produced by adding deionized water and phenol in various concentrations (25–500 mg/L).

### Immobilization methods

Immobilization of TiO_2_ nano powder on concrete surfaces was carried out by four different procedures. Adhesion properties of cement and concrete sealers were applied to fix photocatalyst on pervious concrete surfaces.

#### Slurry method (SM)

The details of this technique are given as follows:

- 20 g/L slurry TiO_2_ was prepared using 25 % (v/v) methanol in deionized water. Methanol would help with the attachment of TiO_2_ to the surface.
- The solution was stirred vigorously for 10 min at 20 °C.
- The slurry was sonicated for 5 min to separate the flocculated TiO_2_ and to obtain more uniform slurry.
- One half of slurry sprayed on fresh concrete to use adhesive properties of cement.
- The coated surface was placed in oven at 100 °C for 2 h to remove the moisture content.
- The rest of the slurry sprayed on hardened concrete and annealed at 450 °C for 2 h to remove any organics from the surface.
- The support was let to dry and was washed with pure water to eliminate the excess of the catalyst.

#### Cement mixed method (CMM)

In this method, 20 g/L TiO_2_ was mixed by cement grout (40 g cement + 20 mL water) and distributed on hardened concrete base by an ordinary brush. After 8 h the concrete surface was placed in oven at 100 °C for 2 h to be dried. The support was washed with pure water to eliminate the excess of the catalyst.

#### Epoxy sealer method (ESM) & Waterproof sealer method (WSM)

In these procedures, the effect of epoxy concrete sealer (Nitofix- Fars Iran Company) and waterproof concrete sealer (Nitotile- Fars Iran Company) for adhesion of TiO_2_ was examined. The details of this technique are as follows:

- 100 mL of the selected concrete sealer mixed with 1000 mL of pure water and stirred vigorously for 10 min at 20 °C.
- Prepared emulsion was distributed on the hardened concrete surface with a trowel and let to dry for 20 min.
- After that time, 20 gr/L of TiO_2_ poured on concrete surface for adhesion on sealer.
- The support was let to dry and was washed with pure water to eliminate the excess of the catalyst.

### Photocatalytic reactor setup

**Fig. 1:**
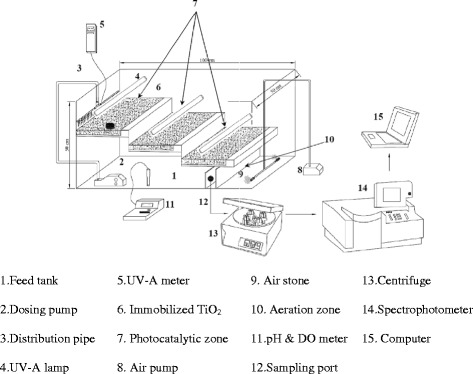
Schematic design of photo-catalyst reactor

The immobilized concrete surfaces were placed in the pilot scale photocatalytic reactor (Fig. 1). A dosing pump was used to feed the phenol-laden wastewater into photocatalytic zone and distributed by a pipe with 1 cm mesh in its length. Reactor hydraulics parameters were controlled by water depth limited to 4 mm. Three concrete surfaces and circulation mode was used to prepare efficient contact time between photocatalyst and wastewater. Dissolved oxygen (DO) and pH was monitored in all the experimental period time by Crison-Oxi45 and Metrohm690, respectively. UV radiation was provided by UV-A and UV-C Philips medium pressure lamps. Samples were taken periodically form the sample ports for phenol concentration analysis. Prior to measurement, the liquid samples were centrifuged by Sigma101 at about 4000 rpm for 10 min to remove detached TiO_2_ particles. The phenol concentrations were measured by colorimetric 4-aminoantipyrine procedure using a Perkin Elmer-Lambda EZ 150 UV/vis spectrophotometer as described in standard methods (2005). The structure and morphology of prepared catalysts and concrete surface were determined using Philips XL30 scanning electron microscope (SEM) followed by AU-coated by sputtering method using a coater sputter (Bal-Tec, Switzerland). All other parameters were determined according to the standard methods (2005). The intermediates determined by GC/Mass (column: chrompack CP-Sil 8 CB 50 m × 250 μm × 0.12 μm). The gas vector used was helium and the detector was a FID. The program of temperature was: detector temperature = 240 °C, injector temperature = 230 °C, oven initial and final temperatures = 105 °C & 190 °C, oven rise 10 °C min^−1^, initial and final time = 2 & 15 min). All other parameters were determined according to the Standard Methods [31].

## Results and discussion

### Characteristics of immobilized concrete surfaces

**Fig. 2:**
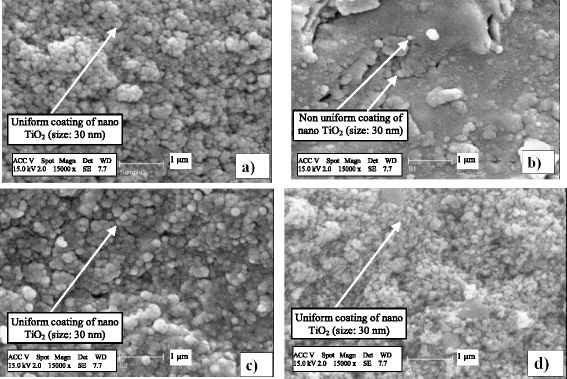
SEM picture and EDX spectrum of TiO_2_ film coated on concrete surface: **a** SM, **b** CMM, **c** ESM, **d** WSM

In all immobilization procedures, SEM and energy dispersive X-ray microanalysis (EDX) were done to confirm the presence of TiO_2_ on concrete surfaces (Fig. 2). Images of non-immobilized surface proved high surface porosity of concrete as a good support for TiO_2_. SEM analysis showed a uniform appearance of TiO_2_ catalyst in ESM, WSM and SM but dispersed coating in CMM.

Mixing TiO_2_ by cement as an adhesive agent caused the minimum level of active surface catalyst in CMM (Fig. 2c). In other methods, catalyst was poured on concrete surface that covered by adhesive agent and consequently uniform TiO_2_ cover was prepared. EDX analysis of TiO_2_ coatings showed no significant levels of noticeable impurities in all immobilizations cases.

### Effect of influent phenol concentration

**Fig. 3:**
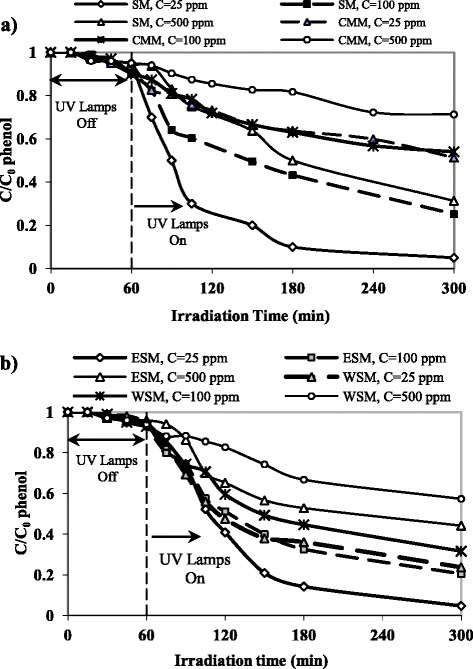
Effect of phenol initial concentration in removal efficiency, **a** SM & CMM, **b** WSM & ESM

Effect of different initial concentrations (C_0_ = 25–500 ppm) in removal efficiency is shown in Fig. 3.

At first, in all experiments the removal efficiency was measured in pH = 7 without UV lamps for 60 min to determine the pollutants adsorption to concrete and phenol volatility. Removal efficiency in these conditions was less than 5 % and stripping of phenol by aeration was negligible because of its very low Henry^’^s constant [32]. Photocatalytic process was then provided by turning on UV lamps and removal efficiency was measured in different retention times. In SM method after 4 h, 95 and 69 % of phenol was degraded in 25 and 500 ppm initial concentration, respectively. Equal degradation was achieved in ESM when C_0_ was 25 ppm but the removal efficiency in WSM was 75 % at the same conditions. The removal efficiency of the CMM was lower than that of other methods. So that after 4 h, 28 % phenol with initial concentration of 500 ppm was degraded. At the same conditions removal efficiency for ESM and WSM were 56 and 43 %, respectively. The results of this study are comparable with similar researches in recent years. For example Chiou et al. (2008) in slurry photo-reactor treated 60 % of 0.52 mM (~49 mg/L) phenol after 3 h and 400 W UV lamp. In other research, Hosseini et al. (2007) reached to 88.3 % phenol degradation after 4 h in initial concentration = 1 mM (~94 mg/L) and UV lamp intensity = 120 W [13].

### Effect of pH solution

**Fig. 4:**
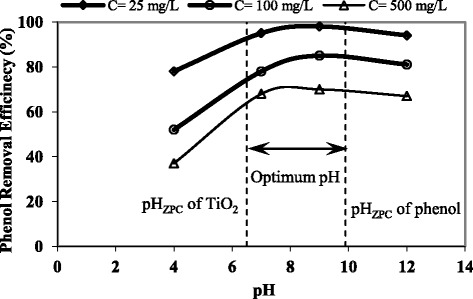
Effect of pH in phenol removal efficiency in ESM

Electrostatic interaction between semiconductor surface, solvent molecules, substrate and charged radicals formed during photocatalytic oxidation is strongly dependent on the pH of the solution [28]. Solution pH dominates photo-degradation process due to the strong pH dependence of many related properties such as the semiconductor’s surface charge state, flat band potential, and the solution dissociation. In alkaline pH, phenol was converted to phenoxide group (remove of H^+^ and creation of negative charge on hydroxyl group) that was more reactive than phenol in a solution [33]. In the other hand, the ionization state of the surface of the photocatalyst can also be protonated and deprotonated under acidic and alkaline conditions. The pH_ZPC_ of Degussa P-25 TiO_2_ used here is 6.5, and phenol is 9.89, respectively [28]. While under acidic conditions, the positive charge of the TiO_2_ surface increases as the pH decreases (TiOH_2_^+^); above pH 6.5 the negative charge at the surface of the TiO_2_ increases with increasing pH (TiO^−^). The optimum pH can be obtained between pH_ZPC_ of photocatalyst and contaminant. Effect of pH in the range of 4–12 on phenol removal efficiency in ESM was evaluated (Fig. 4).

The highest efficiency was observed at pH of 9–12. The difference between phenol removal efficiency at pH of 12 and 4 was determined 39 % in ESM (52 and 86 % removal efficiency) when initial concentration was 100 mg/L and UV-A lamp intensity was equal to 5.33 mW.cm^−2^. The rate of removal efficiency in different pH was similar in different immobilization methods and determined between pH_ZPC_ of phenol and TiO_2_. Several researchers have observed different results about the effect of the pH on the TiO_2_ photocatalytic decomposition of phenol. This discrepancy on the optimum pH may be a function of the various operating conditions considered.

### Effect of immobilization methods

**Fig. 5:**
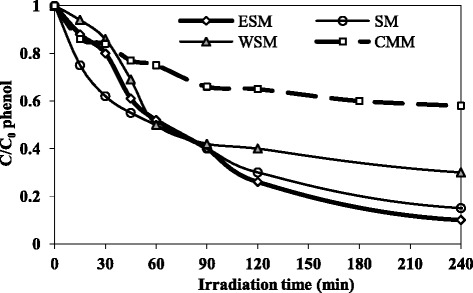
Comparison between phenol removal efficiency by different immobilization methods

The photocatalytic activities for all combinations of coating methods are summarized in Fig. 5. Influent phenol concentration was 50 ppm, pH = 7, UV intensity = 5.33 mW.cm^−2^ and UV lamp distance to concrete surface = 10 cm in these experiments. Results showed ESM had the highest photocatalytic activities so that the removal efficiency of this method was more than 90 % after 4 h. The photocatalytic efficiency of the coating methods was in the following order: ESM > SM > WSM > CMM. The removal efficiency of CMM method after 4 h was 42 % that was the minimum efficiency between other immobilization techniques. The most important reason that proved by SEM-EDX analysis was reduction levels of active surface of nano particles that mixed with cement. While in other methods, TiO_2_ nano particles attached to a concrete top surface, in the CMM nano TiO_2_ particles mixed with cement as cohesive agent and a lot of percents of photoctalyst disappeared. Comparison between ESM and WSM showed that ESM had better performance due to hydrophobic properties of waterproof sealers that decreased connection between contaminants and immobilized pohotocatalyst.

### Effect of UV lamp Intensity

**Fig. 6:**
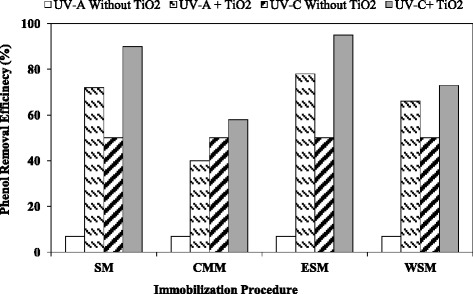
Effect of UV lamp intensity in phenol removal efficiency

The effect of UV lamp intensity on the phenol photo degradation at constant initial concentration (100 ppm) and pH = 7 is presented in Fig. 6. The results showed that phenol removal efficiency increased when the UV lamps intensity promoted. In other words, photocatalytic efficiency in all immobilization methods improved about 50 % when UV-A lamp intensity increased from 4.42 to 8.9 mW.cm^−2^. This is reasonable because the stronger the irradiating UV, the more the UV penetrating. A few differences between phenol degradation since 8.9 and 8.1 UV intensity was confirmed that further increase in UV intensity couldn’t increase the amount of phenol destroyed.

Application of UV-C lamp promoted phenol removal efficiency more than 18 % compared to UV-A lamps at a constant irradiation time. Capability of UV-C lamp in degradation of 50 % of phenol without TiO_2_ photocatalyst showed proper spectral irradiance of UV-A lamp that degraded only 7 % of phenol when photocatalyst was deleted. On the other hand, UV-A lamps had appreciated feature in the photocatalytic process compare to UV-C lamps.

### Effect of long-term use

One of the most important parameters in photocatalytic reactors with immobilized photocatalyst is a reduction in removal efficiency because of TiO_2_ particles detachment and catalyst surface fouling by formation of by-products during the degradation process. This limitation inhibited the application of immobilized procedure as a long term process for wastewater treatment. The influence of long term use in the degradation efficiency has been examined at a constant initial concentration (100 ppm), pH = 7 and UV lamp intensity of 5.33 mW.cm^−2^. Proper connection between TiO_2_ nano particles and concrete surface led to 2 and 3.5 % reduction in phenol removal efficiency for ESM and WSM, respectively. In SM that TiO_2_ poured on concrete surfaces and cement was used as a cohesive agent instead of concrete sealers, the reduction in removal efficiency after 45 times iterations was more than 21 %. Mixing cement by TiO_2_ caused 4 % removal efficiency reduction after 45 times in CMM. In other research, the elimination of some TiO_2_ from the pumice stone surface showed significant decrease of photocatalytic efficiency in long term use of process when SM was used [29].

### Kinetics of the reactions

Langmuir-Hinshelwood kinetic has been used to charac-terize the destruction of many contaminants in different structure of catalyst [34]. The final form of this model can be expressed as Eq. 1:

1 $$ r=-\frac{dC}{dt}=\frac{K_r{K}_s{C}_0}{1+{K}_s{C}_{{}^0}} $$

Where (r) is the rate of the photocatalytic degradation, (C_0_) is the organic concentration, (K_r_) the reaction rate constant, (K_s_) the apparent adsorption constant and (t) is the time of reaction. The term K_s_C_0_ is often negligible when the concentration is low, and the reaction rate can be expressed as pseudo-first-order model as shown in Eq. 2:

2 $$ -\frac{dC}{dt}={K}_r{K}_s{C}_0={K}_{app}{C}_0 $$

Where K_app_ is called apparent first order reaction rate. Integration of the equation yields to Eq. 3:

3 $$ \ln \left(\frac{C}{C_0}\right)=-{K}_{app}t $$

A plot of –ln(C/C_0_) against (t) and slope of linear regression analysis is equal to the value of K_app_. The calculated result indicated that photocatalytic degradation of phenol at the reactions conditions follows a pseudo-first-order kinetics that compared with other researches [13, 35].

Regression coefficients (R^2^) and K_app_ for the photo degradation of phenol at different immobilization method, pH values and initial concentration are shown in Table 1.

**Table 1:** Regression coefficients (R^2^) and K_app_ for phenol photo degradation

C_0_ (ppm)	pH		SM	CMM	ESM	WSM
25	4	R^2^	0.94	0.9	0.96	0.95
		K_app_	0.0044	0.002	0.012	0.003
	7	R^2^	0.99	0.81	0.97	0.93
		K_app_	0.0054	0.0029	0.0135	0.01
	9	R^2^	0.97	0.88	0.95	0.93
		K_app_	0.0061	0.0035	0.0161	0.0055
	12	R^2^	0.97	0.87	0.99	0.96
		K_app_	0.007	0.0041	0.0162	0.006
100	4	R^2^	0.94	0.9	0.99	0.98
		K_app_	0.0044	0.0025	0.0058	0.0032
	7	R^2^	0.94	0.91	0.91	0.9
		K_app_	0.0057	0.0025	0.0073	0.0052
	9	R^2^	0.97	0.89	0.92	0.95
		K_app_	0.0061	0.0033	0.01	0.0058
	12	R^2^	0.97	0.88	0.97	0.95
		K_app_	0.007	0.005	0.012	0.0065
500	4	R^2^	0.86	0.92	0.98	0.99
		K_app_	0.0023	0.001	0.0042	0.002
	7	R^2^	0.99	0.95	0.98	0.95
		K_app_	0.0047	0.0013	0.006	0.0023
	9	R^2^	0.93	0.92	0.99	0.98
		K_app_	0.0057	0.0023	0.0075	0.004
	12	R^2^	0.96	0.88	0.98	0.93
		K_app_	0.0058	0.004	0.0089	0.0052

The results showed that K_app_ increased in all immobilization methods and different initial concentration when pH value changes from 4 to 12. Comparison among different immobilization methods approved that ESM had the highest removal efficiency compared to the other methods that had the most K_app_.

### Phenol photo degradation intermediates

The intermediates of photo degradation of 100 mg/L phenol at pH = 9 and intensity = 8.1 mW.cm^−2^ were obtained by GC/Mass analysis. The results showed phenol hydroxylated via hydroxyl radical attack to different extent forming a variety of intermediates from the very beginning of the reaction. Firstly, aromatic ring (hydroquinone, catechol and benzoquinone) produced and after 60 min, intermediates convert to linear compounds such as oxalic acids and formic acids. Lastly, phenol is completely mineralized to CO_2_ and H_2_O. This can be explained given the fact that adsorbed phenol molecules on TiO_2_ are subject to variable interaction with hydroxyl radicals.

**Fig. 7:**
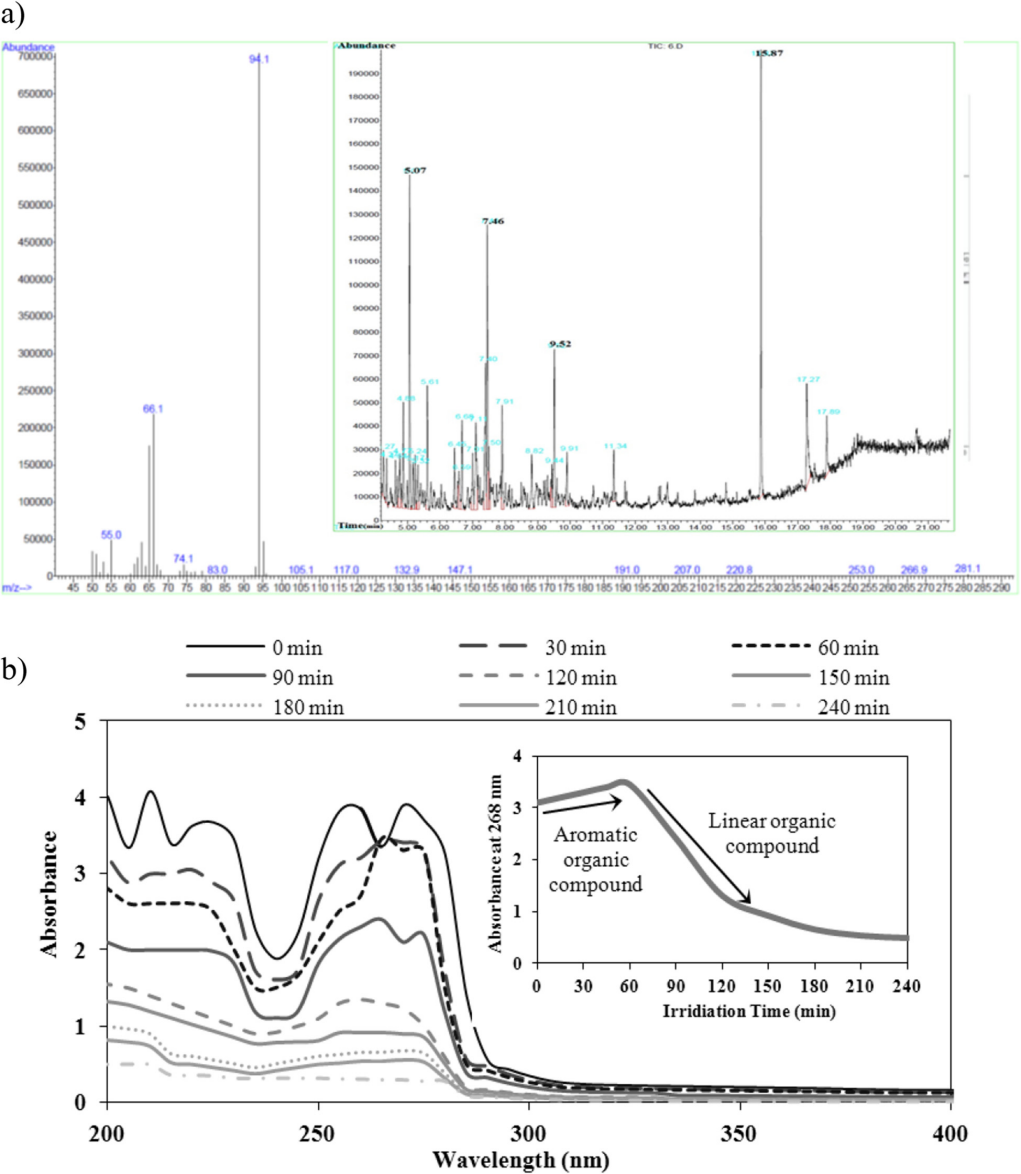
Phenol photo degradation intermediates, **a** GC/Mass analysis, **b** Phenol and intermediates absorbance-time spectrum in 268 nm

Changes of the UV spectrum with respect to destruction of aromatic ring and phenol and variation of the absorbance at 268 nm in different irradiation time are shown in Fig. 7. It can be seen that when the irradiation time increased, maximum peak of the absorption spectrum decreased and finally a spectrum without noticeable peak was observed. At the end, a residual absorbance around 200 nm corresponding to the organic acid formation as final degradation products could be seen.

## Conclusions

In this study, the performances of various coating methods alternatives on concrete surfaces for treating phenolic wastewater were compared. Based on the findings, the following conclusions can be drawn:

1. SEM determination has showed well uniformity of coating processes and EDX spectrum approved nano TiO_2_ on concrete surface.
2. The photocatalytic tests of phenol degradation showed a good photocatalytic activity for TiO_2_.
3. The ESM coating method was found to be the best technique, with the highest photocatalytic activity and adhesion. The removal efficiency of this method was more than 90 % after 4 h.
4. Effect of pH showed that the removal efficiency of phenol increased as pH value increased from 4 to 9 while phenol was converted to phenoxide group.
5. Photocatalytic efficiency in all immobilization methods improved about 50 % when UV lamp intensity increased 2 times.
6. Process kinetics by Langmuir-Hinshelwood model was approved pseudo-first-order reaction for phenol photo degradation.
7. Reduction of phenol removal efficiency was done as a reverse method for evaluation of nano TiO_2_ detachment from concrete surfaces.
8. Results showed that application of concrete sealers had better performance in comparison with SM and CMM that cement was used as a cohesive agent. In WSM and ESM, reduction of removal efficiency was less than 2 and 3.5 % after 45 times iteration of process, respectively.
9. Consequently, concrete due to its special characteristics and large consumption in WWTP as a construction material seems to be an ideal support for the immobilization of TiO_2_ photo-catalysts. Also concrete sealers showed good capability for attachment of TiO_2_ nano particles to concrete surfaces.
